# 

**Published:** 1900-08

**Authors:** 


### CONTENTS.

ORIGINAL COMMUNICATIONS. PAGE

Persistently Retarded Temporary Teeth. By Dr. George S. Allan, New York . 509
Fetor from a Dental Stand-Point. By B. Holly Smith, Baltimore, Md. . . . 514
Actinomycosis about the Mouth. By Charles A. Porter, M.D., Boston, Mass. . 522
Sanitary Condition of the Mouth and Teeth, with a Business Method of obtaining the Same. By Dr. Levi C. Taylor, Hartford, Conn. . . . . . . . . . . 531

ABSTRACTS AND TRANSLATIONS.

The Necessities of a Medical School. By W. W. Keen, M.D., LL.D. . . . . 536

REPORTS OF SOCIETY MEETINGS.

The New York Institute of Stomatology . . . . . . . . . . . . . . . 541
American Academy of Dental Science . . . . . . . . . . . . . . . 552
Academy of Stomatology . . . . . . . . . . . . . . . . . . . 562

EDITORIAL.

What will the Harvest be? . . . . . . . . . . . . . . . . . . 568
The Conventions at Old Point Comfort . . . . . . . . . . . . . . 571

BIBLIOGRAPHY . . . . . . . . . . . . . . . . . . . . . 574

OBITUARY.

Henry H. Burchard, M.D., D.D.S. . . . . . . . . . . . . . . . 576

Charles W. McCall, D.D.S. . . . . . . . . . . . . . . . . . 578

CURRENT NEWS.

Illinois State Dental Society . . . . . . . . . . . . . . . . . 579
Chicago Dental Society . . . . . . . . . . . . . . . . . . . 580
Odontographic Society of Chicago . . . . . . . . . . . . . . . 580

